# “Oh oobe doo, I wanna be like you” associations between physical activity of preschool staff and preschool children

**DOI:** 10.1371/journal.pone.0208001

**Published:** 2018-11-29

**Authors:** Tom Stian Fossdal, Karin Kippe, Bjørn Helge Handegård, Pål Lagestad

**Affiliations:** 1 Faculty of Education and Arts, Nord University, Levanger, Norway; 2 Centre for Child and Adolescent Mental Health, UIT The Arctic University of Norway, Tromsø, Norway; University of Birmingham, UNITED KINGDOM

## Abstract

**Objective:**

Physical activity contributes to prevent serious diseases and ailments, and previous research indicates that lifestyle habits are likely to track from early childhood to adulthood. 90% of Norwegian children aged 1–5 are enrolled in preschools, and preschool staff can play an important role in children’s activity levels. This study’s aim was to identify whether any associations exist between preschool staff’s characteristics (initiative, participation, attitudes, and activity levels) and children’s activity in preschool.

**Method:**

289 children aged 4–6 and 72 preschool staff from 13 randomly selected preschools in a region of Nord-Troendelag, Norway, were enrolled in the study. All participants wore an Actigraph accelerometer for seven consecutive days. Questionnaires were also utilized to identify correlates between preschool staff’s attitudes and initiative in relation to children’s physical activity, in addition to their participation in children’s physical activity. A multilevel analysis, the linear mixed model (LMM), was used to elucidate associations between preschool staff and children’s activity levels.

**Results:**

A significant association was found between preschool staff’s average activity levels during preschool hours and children’s corresponding activity levels during preschool hours (t = 2.57; p = 0.021; f^2^ = 0.013). There were, however, no significant associations identified between the attitudes (t = –0.44; p = 0.67), initiative (t = –0.14; p = 0.89), and participation (t = 0.66; p = 0.52) variables among preschool staff and children’s activity levels during preschool hours.

**Conclusion:**

The study demonstrated that a significant association exists between preschool staff’s aggregated activity levels and 4–6-year-olds’ individual activity levels. However, an observational study is requisite in order to determine whether the association is based on preschool staff’s impact on children’s physical activity or if it is the children that affect the preschool staff’s activity levels, or a combination thereof.

## Introduction

A lack of physical activity is identified as carrying a considerable risk of several diseases [1], and lifestyles characterized by obesity and physical inactivity have a tendency to persist from early childhood to adulthood [2, 3]. Statistics from 2010 show that, globally, approximately 81% of 11–17-year-olds were insufficiently physically active and did not meet the global physical activity guideline (hereafter: PA guideline) of a minimum of 60 min daily MVPA for children [1]. Research also indicates that children are less physically active [4] and spend more time in sedentary activities than their predecessors [5, 6]. Studies have shown that most Norwegian 6-year-olds [7] and Norwegian preschoolers aged 3–4 [8] met the PA guideline of daily physical activity. In contrast, several international studies report that preschoolers are not as active as initially assumed [8–10], and point to the time that children spend indoors as a deleterious factor [11]. Findings that illustrate that children are less physically active than earlier is concerning, as lifestyle behaviors might track from preschool age into adulthood [12]. Indeed, longitudinal studies demonstrate that sedentary time starts to increase from age 3–5 [13] and age 7–9 [14]. Moreover, a cross-sectional study conducted by Goodman et al. [15] found that the total amount of physical activity decreases by an average of 4.2% (3.7% for boys and 4.6% for girls) each year from the age of 5–18.

The Norwegian preschool framework plan emphasizes physical activity, as promoting positive attitudes and actions is considered crucial for children’s perception of physical activity [16]. Adult involvement in play situations and physical activity might, for instance, lead to more recognition for children. This is especially achieved through interaction and collaboration [17], which are essential for staff to promote physical activity and a healthy lifestyle [18]. In this regard, Goldfield, Harvey, Grattan and Adamo [19] assert that physical activity should be initiated as early as possible since children’s activity patterns are more easily influenced by role models’ attitudes. In addition, the foundation for a physically active lifestyle is formed by bodily experiences at a young age [20, 21], in which children should be introduced to physical activity as being enjoyable [22]. These findings could, therefore, support the need to investigate whether pre-schoolers are more or less physically active in preschools where preschool staff initiate and participate in physical activity during preschool hours.

Preschools are considered as an important arena in which to reach as many children as possible, as 90% of Norwegian children aged 1–5 attend a preschool [23]. Additionally, Finn, Johannsen and Specker [24] identified preschools as a major determinant of physical activity, given that more than 50% of the average daily activity counts occurred during children’s preschool hours. Furthermore, a new study using accelerometery among Norwegian preschool staff found that preschool staff, in general, had a high activity level during work [25], whereas preschool staff working with older children (4–6 years old) had the highest activity level [26]. These findings indicate that preschool constitutes an arena in which children can meet and interact with adults who have high activity levels. An appropriate follow-up question is, therefore, whether or not preschool staff’s activity levels during work affect children’s activity levels when they are in preschool.

The extant growing interest in researching preschoolers’ activity levels [27–29] seems especially important, as some children do not naturally participate in play because it might necessitate a certain social competence [16]. In a Danish study of preschoolers’ barriers to physical activity, Nielsen and Eiberg [30] found a correlation between previously satisfying experiences with physical activity, self-esteem, and increased welfare in social environments. This is in accordance with findings reported by Bower et al. [31], who reported that children had a higher activity level if they attended a preschool with a supportive environment where preschool staff participated in their play and gave positive prompts regarding being physically active. These findings also support the view of Sørensen [22], who suggests that preschool staff should engage in physical activity with children, in which physical activity is expressed as fun, instead of a duty, through verbal instructions. In other words, the way that preschool staff and adults generally respond to and confirm children’s activity is crucial to how children perceive themselves [16].

Nevertheless, findings from Sansolios and Mikkelsen [20] revealed that some preschool staff felt pressured to assume all of the responsibility for initiating children’s health habits, a practice with which they did not agree. However, it should also be noted that some researchers [32, 33] have reported that attitudes and actions do not always correspond. In preschool, this is seen as preschool staff acting in terms of their own preferences in spontaneous reactions, rather than following others’ expectations of what to do [34]. Copeland et al. [35] demonstrated, thus, that preschool staff held the key to children’s physical activity, as they were the ones to decide what opportunities children should have to be physically active, in addition to the degree of involvement or dedication that they should have with the children. Regarding this, Eagly and Chaiken [36] claimed that attitudes are evaluated on the basis of a favor–disfavor relationship. Consequently, an interesting aspect is how preschool staff attitudes affect children’s physical activity level.

Several studies have found positive effects of adult-structured activities in preschools [11, 37, 38]. For example, Brown, Googe, McIver and Rathel [39] claim that, in particular, engagement in terms of encouragement, praise, and recognition may affect children’s activity levels in a positive manner. This is supported by Gubbels et al. [40] and Brown et al. [11], who argue that positive encouragement and involvement by preschool staff is associated with higher activity levels in children. Preschool staff’s individual attitudes and behavior may, therefore, play an essential role in promoting children’s physical activity [18].

Considering findings in the extant literature, it seems crucial to identify factors in the activity itself that can lead children to increase their time being physically active. However, limited research exists that addresses the importance of preschool staff’s attitudes, initiative, and participation in physical activities along with children. Qualitative methods seem to constitute the most frequently utilized methodology. No study has yet explicitly investigated the extent to which preschool staff’s expressed attitudes towards physical activity are related to spontaneous activities. Moreover, no researchers have yet studied children’s and preschool staff’s activity levels using accelerometery to identify associations between the physical activity level of preschool staff and children’s physical activity level in preschool. Since preschool staff’s role in children’s physical activity has been objectively measured only in intervention studies, a clear need exists for researching preschool staff’s attitudes, participation, and initiative along with children in spontaneous activities. This may lead to a greater awareness of the importance of preschool staff’s initiation of and/or participation in children’s physical activity. Accordingly, the aim of this study was to identify whether any associations exist between children’s activity in preschool, and preschool staff’s characteristics, controlling for children’s activity levels during leisure time. The preschool staff’s characteristics were operationalized as follows: (a) activity levels during preschool hours; (b) attitudes towards children’s physical activity in preschool; (c) willingness to take the initiative in children’s physical activity in preschool; and (d) participation in children’s physical activity during preschool hours.

## Methods

The present study was conducted in collaboration with a larger Ph.D. research project (unpublished) that used accelerometers, questionnaires, interviews, and observations. However, as the aim of the present study did not comprise all aspects of the data collection, only accelerometer data and questionnaire data were included.

### Subjects and procedures

No power analysis was made before the study, but we opted for 300 children, and we sampled preschools until we had reached this number of children. Independently of size and type of preschool, 13 preschools were therefore randomly selected, including all of the 122 preschools from four counsils in Nord-Troendelag, Norway. All preschools agreed to participate in the study—a response rate of 100%. A condition for participation was that both staff and children were full-time in preschool, including that staff were with the children enrolled in the present study for the entire week. Of 364 children aged of 4–6 attending full-time in the 13 preschools, 289 children (145 boys and 144 girls) volunteered to participate by the approval of their primary guardian, yielding a response rate of 79.40%. All of the 72 preschool staff (57 women and 15 men) who worked mainly with the children aged 4–6 agreed to participate. The preschool staff were kept constant to each group of children. The distribution of sexes among children and adults reflects the natural sex distribution in preschools (see Table 1 and Table 2).

**Table 1:** Descriptive characteristics of children (age 4–6): min in MVPA and fulfilment of the global PA guideline.

	Boys (Mean ±SD)	Girls (Mean ± SD)
Sample size (n)	125	119
MVPA Preschool hours (min)	61.7 ± 18.3	55.1 ± 17.3
MVPA Leisure time weekdays (min)	33.6 ± 12.6	30.8 ± 12.8
MVPA Weekend (min)	75.6 ± 31.5	69.3 ± 27.9
PA guideline Met (%) Met during preschool hours (%) Not met (%)	89.6 45.6 10.4	78.2 33.6 21.8

**Table 2:** Descriptive characteristics of preschool staff: min in MVPA and fulfilment of the global PA guideline.

Sample size (n)	64
Age MVPA Preschool hours (min)	39 ± 11.3 17.3 ± 13
MVPA Leisure time weekdays (min)	16.1 ± 13.1
MVPA Weekend (min)	32.3 ± 25.5
Initiative	3.6 ± 0.5
Participation	3.7 ± 0.5
Attitudes	4.6 ± 1
PA guideline Met (%) Met during preschool hours (%) Met with 10 min bouts (%) Met during preschool hours with 10 min bouts (%)	68.7 37.5 38.9 4.8

Preschool staff and parents received written and oral information about the procedures and ethical standards for testing related to sports science prior to signing the written consent form. Preschool staff and parents were also informed about the voluntary nature of the study. Accelerometer data and questionnaire data were collected during five consecutive weeks from the middle of May until the end of June in 2017. During the data collection, participants (or their primary guardian) received an SMS each morning reminding them to wear the accelerometer. The study was approved by the Norwegian Social Science Data Services (NSD).

### Accelerometery

During the last two decades, researchers have tended to use more objective measurements in order to describe participants’ intensity as metabolic equivalents (METs) [41, 42], where 1 MET is defined as the resting energy expenditure. Moderate activities equate to 3–6 METs, and vigorous activity is considered to have ≥ 6 METs [43, 44]. This is due to the definition of physical activity as any muscular activity that increases energy expenditure [45, 46]. Several researchers seem to agree that calorimetric- (including DLW) validated accelerometers may constitute the most promising method to capture physical activity in free-living situations [47–49]. This is because direct observation is imprecise in identifying intensities and levels of energy expenditure during physical activity [50].

Accelerometers can detect intensity, frequency, and duration of both adults’ and children’s physical activity [30, 48], in addition to inactivity estimates [51]. Accelerometers also filter out other noise that is beyond normal human movement [7], such as from electrical devices or vibration from transport in motor vehicles [52]. Furthermore, accelerometers decrease subjectivity [53] and eliminate certain biases, such as social desirability and recall problems [51]. Raw data output produced from accelerometers is expressed as counts per minute (CPM), which refers to all acceleration to which the accelerometer has been exposed, divided by the number of minutes that the accelerometer has been used [7, 54]. However, in order to capture as precise data as possible, counts are summed during user-defined epochs and classified as various intensities (i.e., sedentary, light, moderate, and vigorous) of physical activity based on categorized count thresholds or cut-offs [55, 56].

Actigraph GT1M accelerometers (ActiGraph, Fort Walton Beach, FL, U.S.A.) were assessed to objectively measure preschool staff and 4–6-year-olds’ physical activity over seven consecutive days. Such a strategy is recommended by several researchers [57–59], and the same type of accelerometer and length of study were also applied in a large population study of Norwegian 6-year-olds [7]. The accelerometer had to be placed at the participant’s right hip, which is recommended by Ainsworth, Cahalin [45]. The participants were required to wear the accelerometer every day except during sleep, showering, or other activities involving water. The Actigraph GT1M is validated and reliability-tested for determining physical activity levels for adults [48], children aged 0–5 [60, 61], and against the global health recommendation standard [62].

For initializing and data reduction, Actilife v6.13.3 (ActiGraph, LLC, Pensacola, FL, U.S.A.) was utilized. Accelerometers were set to start recording at 6 a.m. the day after they were distributed and put on, in an effort to counteract the Hawthorne effect [63]. In addition, they were programmed to save data in two different epochs (time intervals), as children tend to spend more time in sporadic and intermittent physical activity than adults [54, 56, 60]. Researchers have therefore recommended 15 s epochs or less when monitoring children, and 60 s epochs for adults [60], whereas the present study chose to use 10 s epochs for children aged 4–6 and 60 s epochs for preschool staff [7, 64]. This was important in order to be able to compare the findings with other large Norwegian population studies of children and adults that include accelerometer data.

Count thresholds for the various intensities were defined following extant Norwegian population studies. Activity with less than 100 CPM was interpreted as sedentary, while light activity was defined as 100–1999 CPM for children [7] and 100–2019 for adults [64]. Furthermore, physical activity between 2000 and 5998 CPM for children [7] and 2020–5998 CPM for adults was considered as moderate intensity [64], requiring 3–6 times as much energy as the resting energy expenditure. The count threshold for vigorous activity was defined as 5999 CPM for both adults and children [7, 64], and requires more than 6 METs [41]. These differences in intensity cut-offs are, according to Troiano et al. [65], due to adjusting for children’s and youths’ higher resting energy expenditure.

Valid days required at least 480 min of daily recorded activity, whereas sequences of 60 min or more for preschool staff [64] or 20 min or more for children with consecutive zero counts, were interpreted as non-wear time and omitted [7]. In accordance with the test protocols of Kolle et al. [7] and Anderssen et al. [64], preschool staff were required to have at least three valid days, while children needed only two (because more days are needed among adults to obtain reliable and validated activity levels), in order to be included in the study. Data between 00:00 and 05:59 a.m. were excluded due to instructions regarding no accelerometer-wearing during sleep. Wear-time was categorized as follows: (a) preschool hours (8 a.m.–3:29 p.m.); (b) leisure time on weekdays (6 a.m.–7:59 a.m. and 3:30 p.m.–11.59 p.m.); and (c) weekends (06 a.m.–11:59 p.m.). A total of 244 children and 64 preschool staff had valid accelerometer data, yielding a response rate of, respectively, 84.4% for children and 88.8% for preschool staff.

### Questionnaires

The main purpose of using self-reported questionnaires was to identify preschool staff’s: (a) attitudes towards physical activity, both for themselves and children; (b) physical activity habits concerning both leisure time and work; and (c) climate for prompting physical activity. Nonetheless, preschool staff were advised to fill out the questionnaire at the end of the week, as self-report questionnaires impose demands on respondents’ memory and abilities to recall physical activity [66]. 68 preschool staff completed the questionnaire, yielding a response rate of 94.4%.

### Statistical analysis

All calculations, except for analyzing effect size, were performed in SPSS statistical software version 23 (IBM SPSS, Chicago, IL, U.S.A.). Five questions, respectively, from the questionnaire that concerned the concept of initiative were computed into an initiative variable, and four questions concerning the concept of participation were computed into a participation variable. However, only one variable was considered to be directly related to preschool staff’s attitudes towards children’s physical activity in preschool (see Table 3).

**Table 3:** Variables concerning the concept of attitude, initiative and participation, with numbers and descriptions of questions with reply options (^a^^,^^b^^,^^c^).

**Attitudes**
1. To which extent is it important that children are physically active at least one hour per day? ^a^
**Initiative**
1. When you are with the children, how often do you suggest/initiate physical activities for the children during an average day in preschool? ^b^
2. If you notice one or several children that are not physically active, how do you respond to this? (answer the statements below based on the extent of agreement): Provide children guidance and suggestions for how they can play in physical activity. ^c^
3. If you notice one or several children that are not physically active, how do you respond to this? (answer the statements below based on the extent of agreement): Initiate physical activities for the children. ^c^
4. If children initiate physical activity by themselves, how do you usually respond to this? (answer the statements below based on the extent of agreement): Provide children guidance and suggestions during the activity. ^c^
5. If children initiate physical activity by themselves, how do you usually respond to this? (answer the statements below based on the extent of agreement): Provide children guidance and suggestions when the activity is ending. ^c^
**Participation**
1. When you are with the children, how often do you participate in children’s physical activity during an average day in preschool? ^b^
2. If other preschool staff initiate children’s physical activity when you are present, how often do you participate in these during an average day in preschool? ^b^
3. If you notice one or several children that are not physically active, how do you respond to this? (answer the statements below based on the extent of agreement): Participate in children’s physical play along with the children. ^c^
4. If children initiate physical activity by themselves, how do you usually respond to this? (answer the statements below based on the extent of agreement): Participate along with the children. ^c^

^a^ Unimportant, less important, neither important nor unimportant, a bit important, very important (valued from 1–5).

^b^ Never, seldom, occasionally, usually, all the time (valued from 1–5).

^c^ Totally disagree, partially disagree, neither disagree nor agree, partially agree, totally agree (valued from 1–5).

Since children are nested in different preschools, data were characterized as hierarchical, as a child’s activity level might be affected by other children’s activity levels in the same specific preschool. Consequently, a multilevel analysis (linear mixed model (LMM) analysis) was used to examine associations between children’s activity levels and preschool staff’s activity levels, as it can handle data dependency that occurs in such cases. Using residual analysis via inspection of residual plots, the assumptions of the linear mixed model (normally distributed residual, linearity and homogeneity of variance) showed no obvious violations. To measure activity level, the average MVPA per day was preferred, as MVPA is, according to Kolle et al. [7], linked directly to the global PA guideline. Moreover, a multilevel analysis has been considered as a suitable method to capture social contexts with several levels [67].

Preschool staff’s accelerometer data were aggregated into average activity level among staff in each specific preschool, as children were not in contact with only one employee, but all of the preschool staff. Therefore, it was assumed that preschool staff’s average MVPA reflects their impact on children, as some of the staff might be very active while others are less active in engaging children in physical activity, whereby both behaviors may affect children in different ways. The association between children’s preschool MVPA and preschool staff’s MVPA during preschool hours was also controlled for other predictors (i.e., children’s MVPA at leisure time, preschool staff’s attitudes, preschool staff’s initiation, and preschool staff’s participation) in the same LMM analysis. The rationale for controlling for children’s activity levels during leisure time, is that we wanted to assess the unique association between children’s and staff’s MVPA within preschools, and not confound this association with children’s general activity level. In addition, all variables were added in one step. Since the main independent variable was staff activity level, and the other variables played a role as covariates, a stepwise procedure was not appropriate.

Stata statistical software version 15 (StataCorp LLC, Texas, U.S.A.) was performed to measure local effect size, following the procedures described by Bruin [68]. As a measure of local effect size, i.e., the effect of one of the variables in the model in the context of a multivariable linear mixed model, Cohen’s *f*^2^ was computed [69]. Cohen [70] indicated approximately that f^2^ = 0.02 reflects a typical small effect, f^2^ = 0.15 a typical medium effect, and f^2^ = 0.35 a typical large effect.

## Results

**Fig 1:**
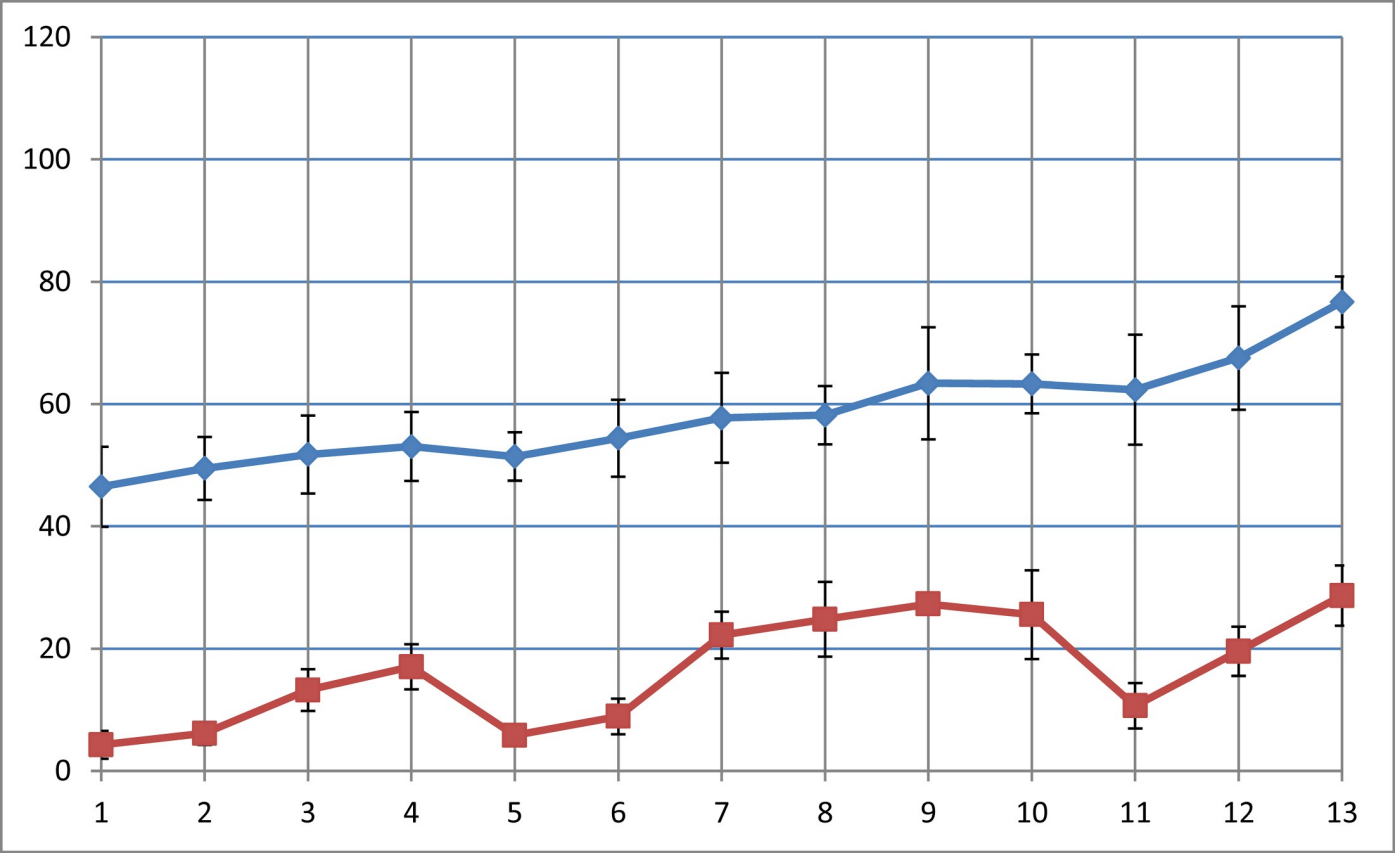
Associations between children's and preschool staff’s average objectively measured MVPA during preschool.

**Fig 2:**
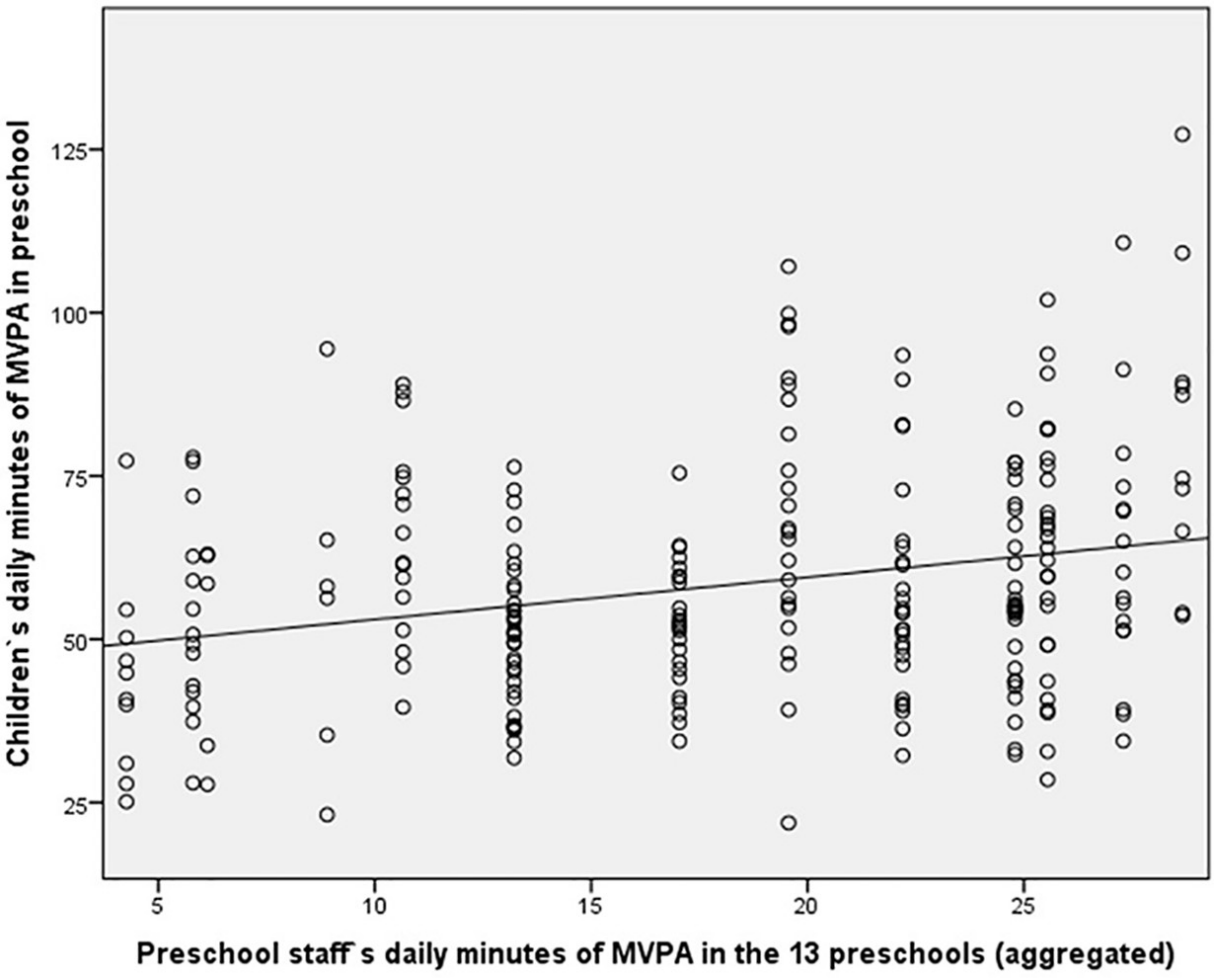
Associations between preschool staff’s aggregated MVPA and children`s individual average MVPA during preschool hours.

The LMM analysis showed that a significant association exists between preschool staff’s average activity levels during preschool hours and children’s activity levels during preschool hours (t = 2.57; p = 0.021; f^2^ = 0.013). According to Cohen’s (1988) definition of typical small, medium and large effects, the size of this f^2^ can be considered to be small. However, this finding is illustrated with two figures in order to show preschool staff’s aggregated data during preschool hours with children’s predicted MVPA during preschool hours in each preschool (Fig 1), and children’s individual average MVPA during preschool hours linked to the preschool that they are attending (Fig 2).

Fig 1 shows that a difference exists between preschools, and thus LMM is requisite. Despite an LMM analysis on an individual level, Fig 1 may be informative in gaining a visual impression of how the average in both the staff’s and children’s MVPA in each specific preschool correspond, while Fig 2 shows the individual variation in MVPA among the children in the 13 preschools. Furthermore, although Fig 2 reveals great differences between children’s activity levels on an individual level, a tendency for children’s activity levels to increase along with the preschool staff’s aggregated activity levels in each specific preschool is seen in both Figs 1 and 2. There were, however, no significant associations between preschool staff’s attitudes (t = –0.44; p = 0.67), initiative (t = –0.14; p = 0.89), participation (t = 0.66; p = 0.52), and children’s activity levels during preschool hours. Furthermore, the intraclass correlation for the MVPA preschool hours variable was 0.195, indicating that 19.5% of the total variance in MVPA preschool hours resided between preschool means.

## Discussion

The first main finding demonstrates the importance of active employees in preschool, as a significant association exists between preschool staff’s average activity levels and children’s activity levels during preschool hours, controlled for other predictors. In addition, children spent more time in MVPA in preschool than in their leisure time during weekdays, which is similar to findings reported by Finn et al. [24], in which preschoolers’ accelerometer counts from 9 a.m. to 5 p.m. accounted for more than 50% of their daily average counts, and in which the preschool was identified as a major determinant of children’s physical activity. This is in contrast to findings reported by Hinkley et al. [8], which demonstrated that boys and girls in preschool were more physically active outside preschool hours on weekdays, using the same accelerometer type and statistical test as the present study. However, the differences were very small in the study by Hinkley et al. [8], and neither Finn et al. [24] nor Hinkley et al. [8] provide information about how much time children spent at preschool or at leisure. While it seems that the preschool children in the present study spend twice as much time in preschool than outside preschool hours, there is no indication that this has been taken into account in the studies by Hinkley et al. [8] and Finn et al. [24].

Moreover, children spent, on average, more minutes in MVPA during weekdays than on the weekends, and since most of the MVPA during weekdays was achieved in preschool, this finding indicates that preschool is an important arena for children’s daily physical activity. In addition, other studies have found that preschool staff have generally high activity levels during work [25]. Those who work with children from 4–6 years old have been shown to have the highest activity levels, at 56 min in MVPA per day [26], which is much more than other Norwegian women (34.3 min MVPA per day) and men (36.5 min MVPA per day) in the same age group as the preschool staff in the present study [71].

However, an essential question is whether the association between preschool staff and children is based on preschool staff’s impact on children’s physical activity, or if it is the children that initiate all of the activity in the preschools and affect the preschool staff’s activity levels, or a combination thereof.

The second main finding from the LMM analysis was that no significant associations existed between the preschool staff’s initiation, participation and attitudes, and children’s activity levels during preschool hours. However, this could be due to the difficulty in operationalizing the terms of initiation, participation, and attitudes into items in a questionnaire. It may also be the case that the questions may have been inadequate to fully capture the variables to be measured (validity) [72]. In addition, self-reported questionnaires might suffer from certain reliability issues, as they depend heavily on the individual respondent’s own perception, memory, and concentration [66].

Nevertheless, previous research has reported conflicting findings concerning the concept of preschool staff’s initiation, participation, and attitudes in relation to physical activity. Mikkelsen’s [18] self-reported study on 3–5-year-olds’ physical activity found that preschool policy and guidelines, which encourage play and movement, were associated with more children undertaking moderate activity. In addition, he claimed that preschool staff’s individual attitudes and behavior also play an essential role in promoting children’s physical activity [18]. In contrast, Cashmore and Jones [73] demonstrated through interviews that preschool staff considered child-directed play as most valuable for children, and thus were reluctant to interfere. Several researchers have, for this reason, identified portable equipment and toys as a key factor for children’s physical play [31, 40, 74], indicating that adults do not have to interfere as long as children have opportunities to play while they are in motion.

Regarding the participation variable, general agreement among several researchers [11, 29, 40] indicates that positive adult encouragement is critical when preschool staff participate in children’s physical activity. Positive adult encouragement might increase children’s physical activity through perceived sport competence [29] and lead to more recognition for children, especially through interactions and collaboration [17]. In addition, a correlation was identified between previously satisfying experiences with physical activity, self-esteem, and increased welfare in social environments in a Danish study by Nielsen and Eiberg [30]. Their study and other studies [11, 19, 29, 40] indicate that preschool staff might have a crucial impact on children’s activity levels if they provide a supportive environment in which physical activity is prompted regularly. This might contribute to explain the findings in Figs 1 and 2, as preschool staff from preschools with high activity levels might have inspired the children to be more active, or preschool staff chose to be physically active with the children when the children requested this.

Regarding the initiation variable, Copeland et al. [35] reported that the preschool staff in her interview study claimed that they held the key to children’s physical activity. This was because they were the ones to decide what opportunities children should have to be physically active, in addition to the degree of involvement or dedication that they should have with the children. Moreover, findings from a qualitative self-reported study conducted by Sansolios and Mikkelsen [20] revealed that some preschool staff felt pressured to assume all of the responsibility for initiating children’s health habits, a practice with which they did not agree. These findings suggest that major differences exist in preschool staff’s beliefs and behavior regarding their role to initiate children’s play and physical activity.

### Strength and limitations of the study

The present study possesses several advantages. Firstly, it includes a large number of participants, whereby the distribution of children’s sex is more or less equal, reflecting the actual sex distribution in preschools. Moreover, both large and small preschools, in addition to different types of preschools, were included in the study as a result of being randomly selected. This provides a representative sample, as the size and type might differ greatly between preschools. Secondly, to the best of our knowledge, this is the first study to objectively assess both children’s and preschool staff’s physical activity with accelerometers. Objective measurements, such as those obtained with accelerometers, offer a major advantage, as they decrease subjectivity [53] and eliminate certain biases, such as social desirability and recall problems [51]. Furthermore, it provides opportunities to compare findings with other studies, as accelerometers have been widely utilized in the last two decades [75]. The present study’s use of accelerometery is based on high-quality standard procedures and justified by the following two reasons: (a) accelerometers are demonstrated to correspond well with energy expenditure related to free-living activities [49]; and (b) the Actigraph GT1M is validity- and reliability-tested for researching physical activity levels for adults [48], children aged 0–5 [60, 61], and against the global PA guideline [62]. Finally, it should be noted that the present study used a rather advanced statistical analysis in LMM. Such a strategy possesses certain advantages, as it handles data dependency that occurs when participants are nested within groups, in addition to the fact that a multilevel analysis is considered as a suitable method to capture social contexts with several levels [67].

Nevertheless, the present study is not without limitations. Some information about child-teacher associations in physical activity levels may have been lost due to aggregating teacher activity levels within the preschools. In addition, the sample includes many women and few men among the preschool staff. This may have affected the results, depending on how men and women may behave differently, in general, regarding initiation and participation in children’s physical activity. On the other hand, it is well known that the preschool profession is dominated by women, which makes the present sample representative of preschools in general. Another disadvantage concerns the use of questionnaires in order to describe the variables regarding preschool staff’s initiative and attitudes to children’s physical activity, in addition to their participation in child-directed physical activity. Such a strategy might be difficult to operationalize questions with good validity. In addition, as questionnaires rely on respondents’ interpretation of the questions and their ability to recall actions, the questionnaires might have varied accuracy and validity [66]. Furthermore, that a factor analysis was not used in the present study, before computing variables into the concept of preschool staff’s initiation and participation, may constitute a disadvantage. However, due to the recommended minimal sample size for factor analyses, the assumptions for factor analysis were not fulfilled [76]. Furthermore, only one question was used to explain the concept of preschool staff’ attitudes towards children’s physical activity in preschool. However, the question might be important, as it is directly related to the preschool staff’s attitudes concerning children’s physical activity.

Moreover, although accelerometery is considered to be a preferable measurement when assessing physical activity in free-living situations, it is not capable of assessing torso movement accurately when it is attached to the hip [60], which also results in an underestimation of cycling or riding vehicles [53]. This is especially unfortunate, as riding vehicles among other toys has been argued to be important for preschoolers’ physical activity [77]. In addition, due to no water contact, neither swimming nor other activities that involved water that are considered as physical activities were included in the data analysis, which might lead to an error in estimation of the participants’ accelerometer counts.

## Conclusion

To the best of our knowledge, the present study is the first to apply accelerometers as an objective measurement for both children and preschool staff when assessing staff’s impact on children’s physical activity. The findings demonstrate that a significant association exists between preschool staff’s aggregated activity levels and 4–6-year-olds’ individual activity levels. However, there were no significant associations between the concept of preschool staff’s self-reported initiation, participation and attitudes, and children’s activity levels. Consequently, the need to examine these characteristics remains, using a mixed-method design including observation, objective measurements, and more valid measurements of attitudes, initiation, and participation. Future research should also use direct observation to determine whether children’s active play is self-initiated or being prompted or led by preschool staff. This might identify whether children are physically active or inactive by nature, or if they are affected by those who are supervising them. A longitudinal study would also be preferable in order to explain possible side effects from encouraged physical activity in terms of initiation, participation, and general attitudes towards children’s physical activity, by preschool staff and primary guardians.

## Supporting information

S1 File (SAV)
